# Changes in ocular biological parameters after cycloplegia based on dioptre, age and sex

**DOI:** 10.1038/s41598-022-25462-1

**Published:** 2022-12-28

**Authors:** Yulin Tao, Xiaokang Cheng, Can Ouyang, Xiaoyong Qu, Wenjiang Liao, Qiong Zhou, Jun Ouyang

**Affiliations:** 1 Department of Ophthalmology, Jiujiang No 1 Peoples Hospital, Affiliated Jiujiang Hospital of Nanchang University, 48 South Taling Road, Jiujiang, 332000 Jiangxi China; 2 First Affiliated Hospital of Nanchang University, Jiangxi Center of National Ocular Disease Clinical Research Center, 17 Yongwai Main Street, Nanchang, 330000 Jiangxi China

**Keywords:** Diseases, Health care, Medical research

## Abstract

The effects of cycloplegia on ocular biological parameters in children have been extensively studied, but few studies have compared these parameters between different refractive states, ages, and sexes. Therefore, the purpose of this study was to investigate the changes in ocular biometry before and after cycloplegia in different groups based on dioptre, age and sex. We examined a total of 2049 participants in this cross-sectional study. A comprehensive eye examination was conducted before cycloplegia. Cycloplegia was implemented with the application of atropine or tropicamide. Ocular biological parameters were evaluated after cycloplegia, including axial length (AL), mean keratometry (K), flat keratometry (K1), steep keratometry (K2), central corneal thickness (CCT), anterior chamber depth (ACD) and white-to-white (WTW) distance. All the participants were categorized based on dioptre, age and sex. Statistical analysis was performed with paired t tests and Wilcoxon signed-rank tests. Regarding dioptre, AL was found to be increased significantly in the Fs, Ast and FA (*p* < 0.05) postcycloplegia groups. We observed significant increases in K, K1, K2 and ACD in the Fs group (*p* < 0.05) after cycloplegia. Regarding age, we found significant increases in AL, CCT and ACD in group 1 (*p* < 0.05), but AL decreased significantly in groups 2 and 3 (*p* < 0.05) postcycloplegia. There were no significant changes found in K, K1 and K2 in the three groups after cycloplegia (*p* > 0.05). Regarding sex, AL and WTW were found to decrease significantly among males and increase significantly among females (*p* < 0.05) postcycloplegia, while K, K1 and K2 showed the opposite trends. This study showed that there were differences in some ocular biological parameters after cycloplegia across different groups; in particular, there were significant differences in AL, CCT and ACD. Attention should be devoted to the influence of cycloplegia in clinical work.

## Introduction

Short-sightedness is caused by a slight elongation of the eyeball, which means that light is focused only in front of the retina rather than on the retina. In the past 50 years, the rate of myopia in East Asian countries has increased sharply^1^. Myopia, especially high myopia, seriously threatens people's physical and mental health and can even lead to blindness. Approximately 2 billion people have myopia, accounting for approximately 28.3% of the global population^2^. Additionally, approximately 277 million people have high myopia, accounting for approximately 4.0% of the worldwide population^2^. It is estimated that by 2050, the number of individuals with myopia will increase to 4.76 billion, accounting for approximately 49.8% of the global population, and nearly 1 billion individuals will have high myopia^2,3^. Ocular disorders among children and adolescents have become a serious social problem in China, and refraction is the primary cause of vision loss in these age groups^4–6^. Therefore, the accurate diagnosis and appropriate treatment of myopia are increasingly important.

With the increasing number of myopic patients, an increasing number of children require orthokeratology, which highlights the great importance of the accuracy of preoperative optometry^7–9^. In addition, due to the increasing number of patients undergoing corneal refractive surgery, such as transepithelial photorefractive keratectomy (T-PRK), laser in situ keratomileusis (LASIK) and laser subepithelial keratomileusis (LASEK), as well as people's stringent requirements for visual quality after the surgery, it is important to accurately evaluate patients' refractive parameters before the operation^10–12^. Cycloplegia optometry is the internationally recognized and the most important method for the diagnosis of ametropia in children and adolescents^13–15^. However, the parameters of the anterior segment can be altered after the application of cycloplegia, including refractive state^16–19^, axial length (AL)^17,19–22^, corneal curvature^23–25^, central corneal thickness (CCT)^16,21,22,26–28^, anterior chamber depth (ACD)^17,19,21–26,29–31^, anterior chamber volume (ACV)^26,30^, and white-to-white (WTW) distance^22,31^. Some researchers have found that significant changes occur in higher-order aberrations (HOAs)^18^, subfoveal choroidal thickness (ChT)^13,20^ and lens thickness^21,30^ among children or adults after the drop in cycloplegia in the eye. Bagheri A and some other researchers found that cycloplegia causes a hyperopic shift^16–19^. Numerous studies have shown that ACD increases after cycloplegia^21,22,30^. All the parameters from the abovementioned studies showed huge differences except ACD, ACV, hyperopic shift, ChT, HOAs and lens thickness. The change in refractive state and anterior segment parameters caused by ciliary paralysis directly affects the diagnosis and treatment of ametropia. Corneal curvature, CCT and ACD are also important indices for the preoperative evaluation of many kinds of ophthalmic surgery, including corneal refractive surgery and cataract surgery. There were considerable differences in the results of previous studies on the biological parameters of the important anterior segment, such as AL, corneal curvature and CCT, even though the opposite results were obtained^17,19,21,22^.

At present, there is a lack of large sample studies on the effects of cycloplegia on refractive state, AL, ACD, corneal curvature and CCT in children and adolescents. The effects of mydriasis optometry after cycloplegia on corneal curvature and CCT have yet to be fully elucidated, and it is unclear whether different cycloplegic drugs have different effects on ocular biological parameters. This cross-sectional study compared changes in corneal curvature, CCT, ACD and AL in children and adolescents before and after cycloplegia based on dioptre age, and sex.

## Methods

### Study participants

In this cross-sectional study, we examined the right eyes of 2049 patients with ametropia. This study was conducted in accordance with the Declaration of Helsinki. This study was also approved by the ethics committee of the First People's Hospital of Jiujiang City in China. Furthermore, written informed consent was obtained from each participant of the study at the time of study enrolment. All patients were divided into groups based on dioptre, age, and sex, and we compared the changes in the ophthalmic biological measurement parameters before and after cycloplegia. We examined children aged between 1 and 21 years old (mean age was 8.99 ± 3.233 years) with ametropia in the outpatient clinic, including 1102 males and 947 females. All patients underwent systematic ophthalmological examination, including the eyelid, conjunctiva, cornea, refraction, vision, best-corrected visual acuity (BCVA), functional slit lamp biomicroscopy (FSLB), intraocular pressure (IOP) and fundus examination. The examinations were performed by an ophthalmologist to rule out organic ophthalmopathy. The inclusion criteria were as follows: (1) children and adolescents aged 1 to 21 years old in the ophthalmology clinic and (2) no eye diseases found after systematic eye examination (vision, FSLB, fundus examination, etc.). The exclusion criteria were as follows: (1) suffering from eye diseases; (2) having a history of eye diseases; (3) suffering from chronic diseases such as asthma and heart disease; and (4) wearing corneal contact lenses in the past 1 month.

### Ocular examinations

Ocular examinations were performed in the hospital after patients were screened for abnormal vision when they were at school. Ocular biological characteristics of the subjects were measured before and after pupil dilation with a noncontact apparatus (AL-Scan, NIDEK CO., LTD., Aichi Prefecture, Japan). A single operating ophthalmologist performed the procedure. All patients were instructed to stare at the detected beam during the measurement. The right eye was measured first, and then the left eye was measured. The following parameters were examined only once: mean keratometry (K), flat keratometry (K1), steep keratometry (K2), CCT, ACD and WTW. AL was generated automatically 5 times, and the results were averaged. Software was used to automatically calculate the values of CCT and ACD through image analysis. ACD was measured as the distance from the anterior corneal pole to the anterior lens surface. Computer-based optometry was performed on the subjects before and after cycloplegia using a desktop autorefractor (RM8900; Topcon Corp., Tokyo, Japan), first in the right eye and then the left eye. Measurements were repeated 3 times in each eye, and then the average value was recorded. All the data were artificially extracted from the device and entered into a spreadsheet, and each patient was assigned a unique identification number.

For children with ametropia who were under 7 years of age, preliminarily diagnosed with hyperopia and merged with strabismus or amblyopia, cycloplegia was induced by 1% atropine sulfate eye gel, dropping eyes 3 times a day for 3 days, and taking the medicine again on the morning of optometry. Cycloplegia was considered complete when the pupil was dilated to at least 6 mm and a pupillary light reflex was absent. Tropicamide phenylephrine eye drops (Mydrin-P) were used for the children aged 7–12 years old, adolescents aged over 12 years old who were preliminary diagnosed with hyperopia, and children or adolescents under 16 years old who had a change of dioptre upon revisit. The dropping was administered 4 times continuously for one day and 5 min apart. We evaluated cycloplegia and pupil dilation of the patients after an additional 30 min. When the pupil was dilated to at least 6 mm and a pupillary light reflex was absent, cycloplegia was considered complete. Patients without complete cycloplegia were not included in the subsequent biometric analysis. Eventually, the following indices were evaluated: AL, K, K1, K2, CCT, ACD and WTW.

### Statistical analysis

All descriptive statistics are expressed as the mean ± standard deviation (‾x ± s). The Kolmogorov‒Smirnov test was performed to detect the normality of the data. A paired-samples t test was used for statistical analysis before and after cycloplegia when the continuous data were normally distributed; otherwise, the Wilcoxon signed-rank test was used. Two-tailed *p* values were used in all analyses. The 95% confidence intervals (CIs) were calculated for pairwise comparisons of different paired samples. A *p* value of < 0.05 was considered statistically significant. All statistical analyses were performed in SPSS 22.0 software (IBM Corp., Armonk, New York, USA).

### Ethical approval and consent to participate

The implementation of this study followed the Declaration of Helsinki. This study was also supported by the ethics committee of Jiujiang No 1 Peoples Hospital (Affiliated Jiujiang Hospital of Nanchang University) in China. Permit Number: JJSDYRMYY-YXLL-2022–243.

### Consent to participate

Written informed consent for study participation was obtained from the parents or legal guardians of the participants in this study at the time of study enrolment.

## Results

This study contained a larger sample size than previous studies. A total of 2049 individuals participated in the study, including 1102 males (53.8%) and 947 females (46.2%). One of the patients was excluded due to the incomplete collection of parameters in the eye. Only data from the right eyes of the patients were analysed. The average age of the 2048 included patients was 8.99 ± 3.234 years (range from 1 to 21 years) (Supplementary Table 1). In this study, the biometric parameters of the eyes of patients were as follows: AL, K, K1, K2, CCT, ACD and WTW. Patients were divided into groups based on differences in dioptre, age and sex. The groups had different numbers of patients because there was some missing information due to different levels of cooperation among the patients. The general data of patients in each group, such as the number of patients, sex distribution, and age, is shown in Supplementary Table 1.

### Overall situation

After studying the changes in biometric parameters among the 2048 subjects before and after cycloplegia, we found that there was a significant increase in CCT and ACD after cycloplegia (*p* < 0.05, 95% CI − 11.787 to − 7.728, − 0.094 to − 0.047, respectively) (Table 1). However, AL, K, K1, K2 and WTW did not change significantly in this overall population (*p* > 0.05) (Table 1), which is different from previous studies.

**Table 1:** Statistical analysis of the mean, SD, and 95% CI of ocular biological characteristics of the subjects pre- and postcycloplegia in all individuals.

Parameter	Condition	Min–Max	Median	Mean ± SD	95% CI	*p* value
AL (mm)	Pre-C	17.54–29.04	23.44	23.354 ± 1.635	− 0.113 to 0.083	0.604
	Post-C	17.57–29.04	23.44	23.369 ± 1.642		
K (D)	Pre-C	38.24–48.61	43.24	43.158 ± 1.529	− 0.079 to 0.106	0.588
	Post-C	38.25–48.69	43.22	43.145 ± 1.522		
K1 (D)	Pre-C	36.72–49.66	42.40	42.332 ± 1.530	− 0.063 to 0.122	0.534
	Post-C	36.89–48.01	42.35	42.303 ± 1.512		
K2 (D)	Pre-C	38.88–51.37	44.00	43.987 ± 1.677	− 0.096 to 0.107	0.738
	Post-C	38.70–50.00	44.00	43.981 ± 1.664		
CCT (μm)	Pre-C	421–697	549.00	548.827 ± 31.169	− 11.787 to − 7.728	< 0.001*
	Post-C	426–666	558.00	558.585 ± 32.274		
ACD (mm)	Pre-C	2.55–4.69	3.75	3.705 ± 0.289	− 0.094 to − 0.047	< 0.001*
	Post-C	2.63–4.49	3.79	3.776 ± 0.256		
WTW (mm)	Pre-C	10.00–13.40	11.80	11.830 ± 0.413	− 0.025 to 0.028	0.973
	Post-C	8.20–13.80	11.80	11.829 ± 0.435		

*AL* axial length; K mean keratometry, K1 flat keratometry, K2 steep keratometry, *CCT* central corneal thickness; *ACD* anterior chamber depth; *WTW* white-to-white distance; *D* dioptre; *C* cycloplegia, *SD* standard deviation; *CI* confidence interval.

*p < 0.05 was considered statistically significant.

### Delaminated by dioptre

In this study, we divided the patients into six groups according to their dioptre, as follows: Fs (farsightedness, 122 patients), Ss (shortsightedness, 360 patients), Ast (astigmatism, 171 patients), EmP (emmetropia, 12 patients), FA (farsightedness combined with astigmatism, 661 patients), and SA (shortsightedness combined with astigmatism, 671 patients).

We observed different results across the refractive conditions. AL changed significantly in all groups (*p* < 0.05) except the EmP group (*p* > 0.05): it increased in the Fs, Ast, and FA groups and decreased in the Ss and SA groups (Tables 2, 3). No significant difference was found in any of the parameters in the EmP group, which may be due to the inclusion of an extremely small number of patients (Table 3). In the remaining five groups, we found significant increases in K, K1, K2 and ACD after cycloplegia (*p* < 0.05), whereas CCT and WTW were not changed significantly in the Fs group (*p* > 0.05) (Table 2). In the Ss group, K1 and ACD significantly decreased and K2 and CCT significantly increased postcycloplegia (*p* < 0.05), but K and WTW did not change significantly (*p* > 0.05) (Table 2). In the Ast group, significant increases in CCT and ACD and significant decreases in K and K2 were observed postcycloplegia (*p* < 0.05), while the changes in K1 and WTW were not significant (*p* > 0.05) (Table 2). In the FA group, there was a significant increase in K1, CCT and ACD after cycloplegia (*p* < 0.05), whereas K, K2 and WTW did not change significantly (*p* > 0.05) (Table 3). In the SA group, K and K1 significantly decreased and CCT significantly increased postcycloplegia (*p* < 0.05), but K2, ACD and WTW did not change significantly (*p* > 0.05) (Table 3).

**Table 2:** Statistical analysis of the mean, SD, and 95% CI of ocular biological characteristics of the subjects pre- and postcycloplegia in the Fs, Ss and Ast groups.

Parameter	Condition	Fs			Ss			Ast		
		Mean ± SD	95% CI	*p* value	Mean ± SD	95% CI	*p* value	Mean ± SD	95% CI	*p* value
AL (mm)	Pre-C	21.957 ± 1.200	− 1.695 to − 1.023	< 0.001*	24.393 ± 0.877	0.819 to 1.199	< 0.001*	22.945 ± 0.827	− 0.813 to − 0.214	0.001*
	Post-C	23.316 ± 1.684			23.384 ± 1.618			23.458 ± 1.853		
K (D)	Pre-C	42.510 ± 1.522	− 1.070 to − 0.311	< 0.001*	43.049 ± 1.397	− 0.358 to − 0.059	0.162	43.551 ± 1.484	0.173 to 0.805	0.003*
	Post-C	43.201 ± 1.538			43.198 ± 1.598			43.062 ± 1.556		
K1 (D)	Pre-C	41.956 ± 1.536	− 0.815 to − 0.055	0.025*	42.578 ± 1.382	0.061 to 0.465	0.011*	42.452 ± 1.601	− 0.062 to 0.623	0.108
	Post-C	42.391 ± 1.492			42.315 ± 1.574			42.172 ± 1.565		
K2 (D)	Pre-C	43.064 ± 1.568	− 1.348 to − 0.544	< 0.001*	43.518 ± 1.445	− 0.782 to − 0.328	< 0.001*	44.710 ± 1.626	0.426 to 1.101	< 0.001*
	Post-C	44.010 ± 1.692			44.073 ± 1.754			43.947 ± 1.694		
CCT (μm)	Pre-C	552.644 ± 32.959	− 7.598 to − 9.389	0.919	547.389 ± 31.457	− 17.101 to − 7.360	< 0.001*	547.470 ± 33.178	− 20.698 to − 6.507	< 0.001*
	Post-C	551.748 ± 31.286			559.620 ± 31.298			561.072 ± 31.917		
ACD (mm)	Pre-C	3.438 ± 0.265	− 0.424 to − 0.216	< 0.001*	3.821 ± 0.201	0.021 to 0.114	0.009*	3.669 ± 0.233	− 0.158 to − 0.002	0.045*
	Post-C	3.758 ± 0.274			3.753 ± 0.257			3.762 ± 0.242		
WTW (mm)	Pre-C	11.791 ± 0.396	− 0.152 to 0.083	0.493	11.853 ± 0.399	− 0.023 to 0.095	0.243	11.863 ± 0.383	− 0.053 to 0.114	0.288
	Post-C	11.826 ± 0.483			11.817 ± 0.423			11.833 ± 0.379		

*AL* axial length; K mean keratometry, K1 flat keratometry, K2 steep keratometry, *CCT* central corneal thickness; *ACD* anterior chamber depth; *WTW* white-to-white distance; *D* dioptre; *C* cycloplegia; *SD* standard deviation; *CI* confidence interval.

*p < 0.05 was considered statistically significant.

**Table 3:** Statistical analysis of the mean, SD, and 95% CI of ocular biological characteristics of the subjects pre- and postcycloplegia in the EmP, FA and SA groups.

Parameter	Condition	EmP			FA			SA		
		Mean ± SD	95% CI	*p* value	Mean ± SD	95% CI	*p* value	Mean ± SD	95% CI	*p* value
AL (mm)	Pre-C	23.738 ± 0.623	− 0.800 to 0.593	0.750	21.891 ± 1.117	− 1.534 to − 1.236	< 0.001*	24.625 ± 1.135	1.090 to 1.384	< 0.001*
	Post-C	23.841 ± 1.117			23.276 ± 1.631			23.387 ± 1.611		
K (D)	Pre-C	42.806 ± 1.257	− 1.168 to 0.848	0.733	43.013 ± 1.684	− 0.257 to 0.088	0.337	43.356 ± 1.416	0.041 to 0.352	0.013*
	Post-C	42.966 ± 1.573			43.097 ± 1.533			43.159 ± 1.471		
K1 (D)	Pre-C	42.398 ± 1.191	− 0.916 to 0.982	0.940	41.993 ± 1.666	− 0.465 to − 0.122	0.001*	42.570 ± 1.396	0.115 to 0.427	0.002*
	Post-C	42.365 ± 1.565			42.286 ± 1.516			42.299 ± 1.477		
K2 (D)	Pre-C	43.215 ± 1.343	− 1.479 to 0.796	0.522	44.030 ± 1.830	− 0.061 to 0.315	0.185	44.135 ± 1.544	− 0.046 to 0.292	0.154
	Post-C	43.557 ± 1.670			43.903 ± 1.677			44.012 ± 1.604		
CCT (μm)	Pre-C	566.083 ± 31.725	− 17.226 to 44.393	0.353	551.539 ± 29.608	− 9.007 to − 2.156	0.001*	546.387 ± 31.887	− 17.364 to − 10.120	< 0.001*
	Post-C	552.500 ± 40.006			557.120 ± 30.800			560.130 ± 33.581		
ACD (mm)	Pre-C	3.863 ± 0.184	− 0.111 to 0.621	0.172	3.465 ± 0.299	− 0.354 to − 0.259	< 0.001*	3.830 ± 0.209	0.004 to 0.066	0.065
	Post-C	3.608 ± 0.273			3.771 ± 0.263			3.795 ± 0.254		
WTW (mm)	Pre-C	11.908 ± 0.616	− 0.218 to 0.818	0.229	11.793 ± 0.441	− 0.075 to 0.023	0.462	11.853 ± 0.396	− 0.043 to 0.052	0.722
	Post-C	11.608 ± 0.427			11.819 ± 0.431			11.848 ± 0.452		

*AL* axial length; K mean keratometry, K1 flat keratometry, K2 steep keratometry, *CCT* central corneal thickness; *ACD* anterior chamber depth; *WTW* white-to-white distance; *D* dioptre; *C* cycloplegia; *SD* standard deviation; *CI* confidence interval.

*p < 0.05 was considered statistically significant.

### Delaminated by age

We divided the individuals into three age groups: 1 to 7 years old (group 1, 703 patients), 8 to 14 years old (group 2, 1252 patients), and 15 to 21 years old (group 3, 94 patients). AL increased significantly in group 1 and decreased in groups 2 and 3 (*p* < 0.05) (Table 4). No other parameters were found to be significantly changed in group 3 (*p* > 0.05) (Table 4) except AL. Additionally, K, K1, K2 and WTW were not found to be significantly changed in any of the three groups (Table 4). Significant increases in CCT and ACD were observed in group 1 postcycloplegia (*p* < 0.05). However, in group 2, only CCT increased significantly (*p* < 0.05); the change in ACD was not significant (*p* > 0.05) (Table 4).

**Table 4:** Statistical analysis of the mean, SD, and 95% CI of ocular biological characteristics of the subjects pre- and postcycloplegia in group 1 (age ranging from 1 to 7 years), group 2 (age ranging from 8 to 14 years) and group 3 (age ranging from 15 to 21 years).

Parameter*	Condition	1–7 years			8–14 years			15–21 years		
		Mean ± SD	95% CI	*p* value	Mean ± SD	95% CI	*p* value	Mean ± SD	95% CI	*p* value
AL (mm)	Pre-C	22.205 ± 1.297	− 1.261 to − 0.947	< 0.001*	23.886 ± 1.415	0.362 to 0.593	< 0.001*	24.871 ± 1.771	1.099 to 2.050	< 0.001*
	Post-C	23.309 ± 1.686			23.408 ± 1.611			23.297 ± 1.706		
K (D)	Pre-C	43.129 ± 1.519	− 0.175 to 0.140	0.830	43.164 ± 1.533	− 0.087 to 0.150	0.599	43.310 ± 1.554	− 0.436 to 0.448	0.978
	Post-C	43.146 ± 1.515			43.132 ± 1.524			43.304 ± 1.568		
K1 (D)	Pre-C	42.180 ± 1.511	− 0.307 to 0.009	0.065	42.406 ± 1.534	0.009 to 0.246	0.053	42.489 ± 1.543	− 0.390 to 0.505	0.799
	Post-C	42.329 ± 1.502			42.279 ± 1.512			42.432 ± 1.598		
K2 (D)	Pre-C	44.078 ± 1.697	− 0.058 to 0.287	0.168	43.925 ± 1.659	− 0.181 to 0.078	0.556	43.131 ± 1.738	− 0.530 to 0.443	0.859
	Post-C	43.963 ± 1.643			43.976 ± 1.674			44.174 ± 1.702		
CCT (μm)	Pre-C	546.679 ± 30.597	− 14.736 to − 7.560	< 0.001*	549.624 ± 31.287	− 11.688 to − 6.602	< 0.001*	553.804 ± 32.930	− 17.716 to 2.085	0.120
	Post-C	557.827 ± 33.517			558.769 ± 31.706			561.620 ± 30.654		
ACD (mm)	Pre-C	3.498 ± 0.301	− 0.335 to − 0.236	< 0.001*	3.773 ± 0.250	− 0.026 to 0.026	0.994	3.800 ± 0.230	− 0.070 to 0.117	0.653
	Post-C	3.784 ± 0.258			3.773 ± 0.251			3.776 ± 0.310		
WTW (mm)	Pre-C	11.827 ± 0.437	− 0.052 to 0.042	0.940	11.837 ± 0.401	− 0.026 to 0.041	0.782	11.771 ± 0.389	− 0.150 to 0.083	0.525
	Post-C	11.832 ± 0.428			11.829 ± 0.441			11.804 ± 0.416		

*AL* axial length; K mean keratometry, K1 flat keratometry, K2 steep keratometry, *CCT* central corneal thickness; *ACD* anterior chamber depth; *WTW* white-to-white distance; *D* dioptre; *C* cycloplegia; *SD* standard deviation, *CI* confidence interval.

*p < 0.05 was considered statistically significant.

### Delaminated by sex

We divided the participants into two groups according to their sex: male (group A, 1102 patients) and female (group B, 947 patients). There was a significant decrease in AL in group A and an increase in group B after cycloplegia (*p* < 0.05). K, K1 and K2 increased significantly in group A and decreased in group B postcycloplegia (*p* < 0.05). CCT increased significantly in both groups after cycloplegia (*p* < 0.05). ACD was not changed significantly in group A (*p* > 0.05) but increased significantly in group B after cycloplegia (*p* < 0.05). WTW decreased significantly in group A but increased in group B postcycloplegia (*p* < 0.05) (Table 5).

**Table 5:** Statistical analysis of the mean, SD, and 95% CI of ocular biological characteristics of the subjects pre- and postcycloplegia in group A (males) and group B (females).

Parameter	Condition	Males			Females		
		Mean ± SD	95% CI	*p* value	Mean ± SD	95% CI	*p* value
AL (mm)	Pre-C	23.657 ± 1.656	0.138 to 0.406	< 0.001*	23.002 ± 1.539	− 0.490 to − 0.208	< 0.001*
	Post-C	23.385 ± 1.618			23.351 ± 1.670		
K (D)	Pre-C	42.825 ± 1.503	− 0.416 to − 0.169	< 0.001*	43.547 ± 1.466	0.234 to 0.505	< 0.001*
	Post-C	43.117 ± 1.504			43.177 ± 1.543		
K1 (D)	Pre-C	42.000 ± 1.517	− 0.405 to − 0.158	< 0.001*	42.719 ± 1.452	0.256 to 0.527	< 0.001*
	Post-C	42.282 ± 1.494			42.327 ± 1.533		
K2 (D)	Pre-C	43.647 ± 1.640	− 0.434 to − 0.164	< 0.001*	44.383 ± 1.632	0.211 to 0.511	< 0.001*
	Post-C	43.946 ± 1.643			44.022 ± 1.688		
CCT (μm)	Pre-C	550.957 ± 30.623	− 9.840 to − 4.428	< 0.001*	546.316 ± 31.634	− 15.907 to − 9.796	< 0.001*
	Post-C	558.091 ± 32.515			559.167 ± 31.995		
ACD (mm)	Pre-C	3.757 ± 0.281	− 0.046 to 0.017	0.772	3.644 ± 0.286	− 0.171 to − 0.102	< 0.001*
	Post-C	3.771 ± 0.258			3.781 ± 0.254		
WTW (mm)	Pre-C	11.913 ± 0.414	0.045 to 0.119	< 0.001*	11.734 ± 0.390	− 0.130 to − 0.055	< 0.001*
	Post-C	11.831 ± 0.435			11.826 ± 0.437		

*AL* axial length, K mean keratometry, K1 flat keratometry, K2 steep keratometry, *CCT* central corneal thickness, *ACD* anterior chamber depth; *WTW* white-to-white distance; *D* dioptre; *C* cycloplegia, *SD* standard deviation; *CI* confidence interval.

*p < 0.05 was considered statistically significant.

## Discussion

Among the 2048 patients included in this study, we found that there were no significant changes in AL, K, K1, K2 and WTW after cycloplegia, but CCT and ACD increased significantly. After grouping patients based on their dioptre, age and sex, we found that only CCT and ACD increased significantly. Regarding dioptre, a significant increase in AL was observed in the Fs and Ast groups, while significant decreases were observed in the Ss and SA groups. There were significant increases in K in the Fs group, significant increases in K1 in the Fs and FA groups, and significant increases in K2 in the Fs and Ss groups. There were significant decreases in K in the Ast and SA groups, significant decreases in K1 in the Ss and SA groups, and significant decreases in K2 in the Ast group. There were no significant changes in K in the Ss and FA groups, K1 in the Ast group, or K2 in the FA and SA groups. Regarding age, we found that AL increased prominently after cycloplegia among younger patients. No differences were observed in K, K1 and K2 based on age. Regarding sex, AL decreased among males and increased among females postcycloplegia. The same trend was observed for K, K1 and K2 among males and females. CCT and ACD both increased significantly among males and females, bin the two groups, but the change in ACD among males was an exception. Interestingly, WTW began to show a significant trend of change in the two groups (decreased in the male group and increased in the female group).

A series of correct management strategies for myopia and control strategies can counteract its high prevalence and damage. Early diagnosis and prevention associated with effective therapeutic measures are needed to improve public eye health^2^. In regard to the early diagnosis of myopia, postcycloplegia optometry is an important process that has been recognized by scholars all over the world^4,5,14,32^. Cycloplegia has a certain effect on the biometric parameters of the eye that will further affect the accuracy of optometry and refractive surgery, for example, the calculation of the degree of intraocular lens^22^.

In previous studies, some scholars confirmed that cycloplegia in children could lead to changes in AL, K, CCT and ACD; in fact, many studies have reported increases in ACD^13,17,19–25,27–31,33^. Similar conclusions were obtained in our study, but the results varied based on the groups. Because of the large sample size, only CCT and ACD increased significantly after cycloplegia in all individuals. In general, an increase in CCT will lead to the prolongation of AL, which is contrary to our study. When accommodation of the eye is eliminated by cycloplegia, the crystalline lens becomes thinner and moves posteriorly in myopia compared to emmetropia and hyperopia after cycloplegia^34^. Therefore, AL does not transform with changes in CCT and ACD. Nevertheless, Cheng HC^17^, Bahar A^20^ and Tuncer I^22^ still detected a positive shift, and Ho MC^19^ and Raina UK^21^ detected a negative shift after cycloplegia. Ho MC^19^ pointed out in his research that the mean AL was reduced by 0.023 mm in those with an emmetropic or hyperopic shift, whereas it was elevated by 0.026 mm in those with a myopic shift of the eye (*p* = 0.003). This type of phenomenon of AL in the study of Ho MC^19^ was different from the observation in our study, in which the mean AL increased by 1.359 mm in the Fs group and decreased by 1.009 mm in the Ss group (*p* < 0.001). Although many studies showed no effect of cycloplegia on AL measurement, most have been done on adult eyes. We believe that children with stronger accommodative ability may be more vulnerable to cycloplegia, resulting in a greater change in the average refractive index and a significant impact on the calculation of AL. Many studies have shown that K decreases after cycloplegia^17,23–25^. Similar to previous studies, we found that K, K1 and K2 increased significantly after cycloplegia in the Fs group, and the greatest increase was found in K2. Some of the above studies were conducted in children and on myopic eyes; therefore, we believe that the low ocular tissue rigidity in the groups of patients in these studies is the cause of the results. The weak corneal biomechanics in children suggests that it may be affected by the contraction of the adjacent ciliary muscles. We are confident that the change in corneal curvature is a mechanical response to the relaxation of the ciliary muscle, but this effect was not significant in our study. Unlike ACD, which was increased in almost all previous studies, changes in CCT were different. Bagheri A^16^, Zeng Y^28^ and Tuncer I^22^ found that CCT increased markedly after cycloplegia; in contrast, Palamar M^26^ and Raina UK^21^ found that CCT decreased significantly postcycloplegia. Three hypotheses have been proposed for the increase in CCT after cycloplegia. The first is that eye drops containing benzalkonium chloride as a preservative may damage corneal epithelial and endothelial cells and cause corneal stroma oedema^35^. The second hypothesis is that reflex tearing after eye drops may lead to an overestimation of corneal thickness. In addition, the antimuscarinic effect of cycloplegic agents may inhibit or reduce the secretion of reflex tears, which probably decreases tear film thickness and reduces central corneal thickness measurements^23^. The third hypothesis is related to the possible effects of contraction or relaxation of ciliary muscles on the cornea, which can also lead to changes in corneal curvature. Cycloplegic agents may increase or decrease corneal thickness but mainly increased in our study. As an exception, ACD was slightly reduced in the Ss group. We speculate that this may be related to the decrease in AL in this group. We did not find significant changes in AL, K, K1, K2 and WTW as a whole, which may be due to the increase in sample size and the inclusion of all kinds of refractive error.

Currently, cataract surgery has gradually become a refractive surgery along with the application of high-quality lenses. There are many reasons for the prediction error for refractive state after cataract surgery, such as the selection of the intraocular lens (IOL) power formula and the accuracy of the various devices used to measure the eye (intraoperative aberrometry [IA] included)^36^. In the calculation of intraocular lenses in cataract surgery, both the Hill-radial basis function (RBF) formula and the Kane formula use the AL, K, ACD, CCT, and even biological sex to make their predictions^36^. As mentioned in the formula and IOL studies, ACD is measured from the corneal epithelium to the lens. Surprisingly, AL, CCT and ACD increased markedly in almost all groups, which will obviously have an adverse effect on the calculation of the intraocular lens.

The effects of cycloplegia on biometric parameters also varied across different age groups. Tuncer I^22^ divided patients into three age groups (50–60 years, 30–40 years and 10–20 years). He found that ACD was signally increased postcycloplegia in all groups, and the greatest increase was in the 10- to 20-year-old age group^22^. This is consistent with our findings that ACD was significantly increased after cycloplegia in the 1- to 7-year-old age group, whereas almost no change was observed in the 8- to 14-year-old and 15- to 21-year-old age groups. Özyol P^37^ pointed out that with increasing age, lens sclerosis reduces the elasticity of the lens capsule, increases the size and weight of the lens, and reduces the reaction of the lens to cycloplegic agents. Our results support the hypothesis mentioned above. We also found that AL and CCT increased more with age, which has not been found in previous studies. Huang J^31^ and Tuncer I^22^ reported a significant increase in WTW postcycloplegia, although these changes appeared to be unverifiable^33,37^. Similar to their results, we found that WTW increased significantly among females and decreased among males. Many biometric devices locate the limbus on the basis of the change in the contrast from paler sclera to darker cornea. However, the borderline between them is not very distinct; furthermore, the contrast difference can be influenced by the illumination and quality of the image. Under the circumstances described above, WTW measurements after cycloplegia could be larger than those obtained previously^37^.

Because of the larger sample size of our study, the results of this study are more reliable. Additionally, we grouped patients according to the type of refraction, age and sex for the first time. The results obtained herein were inconsistent with previous findings. The conclusions we obtained have certain reference value. The limitations of our study were that the age group was too young, and this topic remains to be further explored. It was difficult to dilate pupils in emmetropic children due to the worries of parents; consequently, this group did not include more patients for the trial. We applied different cycloplegic drugs in different age stages; the differences between these drugs need to be examined in future studies. This study demonstrated that many biological parameters of the eye changed after cycloplegia, even when grouped by refractive error, age and sex. CCT and ACD revealed significant increases in almost all classified groups. In view of the significance of this study, we hope to include more patients of other ages in future studies.

## Supplementary Information

Supplementary Information.

## Data Availability

All data generated or analysed during this study are included in this published article. All data are available upon request. Requests for the data should be directed to Yulin Tao.
