# Supplementary material for: Changes in ocular biological parameters after cycloplegia based on dioptre, age and sex
Source: Sci Rep. 2022 Dec 28;12:22470. doi: 10.1038/s41598-022-25462-1 (PMC9797549; doi:10.1038/s41598-022-25462-1)
Supplement: Supplementary file 1 — Supplementary Information. [file 41598_2022_25462_MOESM1_ESM.pdf]

Table S1 The distribution of cases, gender and age of differential delamination of groups

	Total number	Missing number	Gender		Age		
			Male	Female	Min-Max	Median	Mean ± SD
Overall	2049		1102	947	1-21	9.00	8.99 ± 3.233
AL/K/K1	2048	1	1101	947	1-21	9.00	8.99 ± 3.234
K2	2047	2	1102	945	1-21	9.00	8.99 ± 3.233
CCT	1968	81	1065	903	1-21	9.00	9.04 ± 3.230
ACD	963	1086	522	441	1-18	10.00	9.61 ± 3.122
WTW	1967	82	1060	907	1-21	9.00	9.07 ± 3.205
Delamination by diopter							
Fs	122		62	60	1-15	6.00	6.68 ± 2.782
AL/K/K1/K2	122	0	62	60	1-15	6.00	6.68 ± 2.782
CCT	115	7	59	56	1-15	6.00	6.74 ± 2.829
ACD	53	69	27	26	3-15	7.00	7.36 ± 2.975
WTW	112	10	58	54	2-15	6.00	6.92 ± 2.732
Ss	360		201	159	3-18	10.00	10.53 ± 2.447
AL/K/K1/K2	360	0	201	159	3-18	10.00	10.53 ± 2.447
CCT	347	13	196	151	3-18	10.00	10.54 ± 2.455
ACD	203	157	113	90	3-18	10.00	10.42 ± 2.455
WTW	350	10	192	158	3-18	10.00	10.56 ± 2.434
Ast	171		91	80	3-17	8.00	7.95 ± 2.626
AL/K/K1	171	0	91	80	3-17	8.00	7.95 ± 2.626
K2	170	1	91	79	3-17	8.00	7.95 ± 2.634
CCT	166	5	88	78	3-17	8.00	7.96 ± 2.636
ACD	61	110	30	31	3-17	9.00	8.66 ± 3.010
WTW	165	6	88	77	3-17	8.00	7.96 ± 2.623
EmP	12		9	3	5-13	9.50	9.58 ± 2.644
AL/K/K1/K2/CCT/WTW	12	0	9	3	5-13	9.50	9.58 ± 2.644
ACD	6	6	6	0	7-13	11.00	10.67 ± 2.422
FA	661		349	312	1-21	7.00	7.15 ± 2.891
AL/K/K1/K2	661	0	349	312	1-21	7.00	7.15 ± 2.891
CCT	631	30	336	295	1-21	7.00	7.20 ± 2.900
ACD	231	430	122	109	1-17	7.00	7.36 ± 2.877
WTW	624	37	330	294	1-21	7.00	7.20 ± 2.880
SA	671		360	311	1-19	11.00	10.77 ± 2.714
AL	671	0	360	311	1-19	11.00	10.77 ± 2.714
K/K1/K2	670	1	359	311	1-19	11.00	10.77 ± 2.716
CCT	648	23	349	299	1-19	11.00	10.82 ± 2.699
ACD	385	286	213	172	4-18	11.00	11.06 ± 2.565
WTW	656	15	356	300	1-19	11.00	10.80 ± 2.711
Delamination by age							
1-7 years	703		364	339	1-7	6.00	5.45 ± 1.353
AL/K/K1/K2	703	0	364	339	1-7	6.00	5.45 ± 1.353
CCT	663	40	346	317	1-7	6.00	5.46 ± 1.351
ACD	242	461	118	124	1-7	6.00	5.55 ± 1.378
WTW	659	44	343	316	1-7	6.00	5.52 ± 1.319
8-14 years	1252		684	568	8-14	10.00	10.48 ± 1.821
AL/K/K1	1251	1	683	568	8-14	10.00	10.48 ± 1.822
K2	1250	2	684	566	8-14	10.00	10.48 ± 1.819
CCT	1213	39	666	547	8-14	10.00	10.49 ± 1.818
ACD	663	589	374	289	8-14	10.00	10.55 ± 1.787
WTW	1215	37	664	551	8-14	10.00	10.49 ± 1.822
15-21 years	94		54	40	15-21	15.00	15.76 ± 1.064
AL/K/K1/K2	94	0	54	40	15-21	15.00	15.76 ± 1.064
CCT	92	2	53	39	15-21	15.00	15.77 ± 1.070
ACD	58	36	30	28	15-18	15.50	15.76 ± 0.885
WTW	93	1	53	40	15-21	15.00	15.76 ± 1.067

Delamination by gender								
Male		1102		1102	0	1-21	9.00	9.12 ± 3.272
	AL/K	1101	1	1101	0	1-21	9.00	9.12 ± 3.273
	K1/K2	1102	0	1102	0	1-21	9.00	9.12 ± 3.272
	CCT	1065	37	1065	0	1-21	9.00	9.15 ± 3.274
	ACD	522	580	522	0	1-17	10.00	9.78 ± 3.085
	WTW	1060	42	1060	0	1-21	9.00	9.20 ± 3.255
Female		947		0	947	1-18	9.00	8.85 ± 3.182
	AL/K	947	0	0	947	1-18	9.00	8.85 ± 3.182
	K1	946	1	0	946	1-18	9.00	8.85 ± 3.183
	K2	945	2	0	945	1-18	9.00	8.84 ± 3.181
	CCT	903	44	0	903	1-18	9.00	8.91 ± 3.174
	ACD	441	506	0	441	2-18	9.00	9.40 ± 3.157
	WTW	907	40	0	907	2-18	9.00	8.93 ± 3.142
